# Chipless RFID Sensors for the Internet of Things: Challenges and Opportunities

**DOI:** 10.3390/s20072135

**Published:** 2020-04-10

**Authors:** Viviana Mulloni, Massimo Donelli

**Affiliations:** 1 Centre for Materials and Microsystems, Fondazione Bruno Kessler, 38123 Trento, Italy; 2 Department of Information Engineering and Computer Science, University of Trento, 38100 Trento, Italy; massimo.donelli@unitn.it

**Keywords:** chipless RFID, microwave systems, electromagnetic propagation, sensing materials, wireless sensors

## Abstract

Radio-frequency identification (RFID) sensors are one of the fundamental components of the internet of things that aims at connecting every physical object to the cloud for the exchange of information. In this framework, chipless RFIDs are a breakthrough technology because they remove the cost associated with the chip, being at the same time printable, passive, low-power and suitable for harsh environments. After the important results achieved with multibit chipless tags, there is a clear motivation and interest to extend the chipless sensing functionality to physical, chemical, structural and environmental parameters. These potentialities triggered a strong interest in the scientific and industrial community towards this type of application. Temperature and humidity sensors, as well as localization, proximity, and structural health prototypes, have already been demonstrated, and many other sensing applications are foreseen soon. In this review, both the different architectural approaches available for this technology and the requirements related to the materials employed for sensing are summarized. Then, the state-of-the-art of categories of sensors and their applications are reported and discussed. Finally, an analysis of the current limitations and possible solution strategies for this technology are given, together with an overview of expected future developments.

## 1. Introduction

In the last few years, we have seen a dramatic diffusion of wireless technologies. More specifically, the technological advancements of recent years have paved the way for the diffusion and the increasing pervasiveness of wireless sensors technologies in the framework of the internet of things (IoT). The IoT is a system composed of heterogeneous devices and organized as a network. Each device (a thing) is provided with unique identifiers (UIDs) and the capability to share information or transfer data over a wireless or wired network without requiring human interaction. Everyday objects, such as a lamp or a washing machine but also animals and people, could be part of an IoT network if they are provided with a unique identifier and are able to transfer data over the network thanks to a radio frequency front end. A thing is a unit element of an IoT system and is able to collect data from its sensors, and then to process and share data toward a wireless channel. To improve the pervasiveness of an IoT system, devices with a high degree of miniaturization, low power and low cost are urgently needed. In such a scenario the use of radiofrequency identification (RFID) is a very interesting and widespread solution to implement IoT systems or networks of distributed sensors. RFIDs are reasonably cheap, are provided with unique identifiers and can be equipped with a variety of sensing capabilities. Because of these characteristics, RFIDs have been successfully adopted in many practical applications such as the tracking of goods and foods in supermarkets and shops, the tracing of books in libraries, in environmental sensing [1] and for many applications in the general field of the IoT. 

While chipped RFIDs are a well-consolidated technology with an enormous and growing market, current research is concentrated on finding solutions providing an advantage in terms of cost and compactness [2]. The best option to reduce the cost of RFIDs is an approach that eliminates the use of the chip, producing a device that is therefore called “chipless RFID” [3,4]. Such a device can be equipped with sensing capabilities while remaining extremely cheap, printable, and suitable for mass production and harsh environments [5]. Moreover, it can be made disposable or biodegradable [6] using proper materials to fabricate it. 

For all these reasons, in the last few years, the scientific literature paid large attention to these type of sensors.

**Figure 1:**
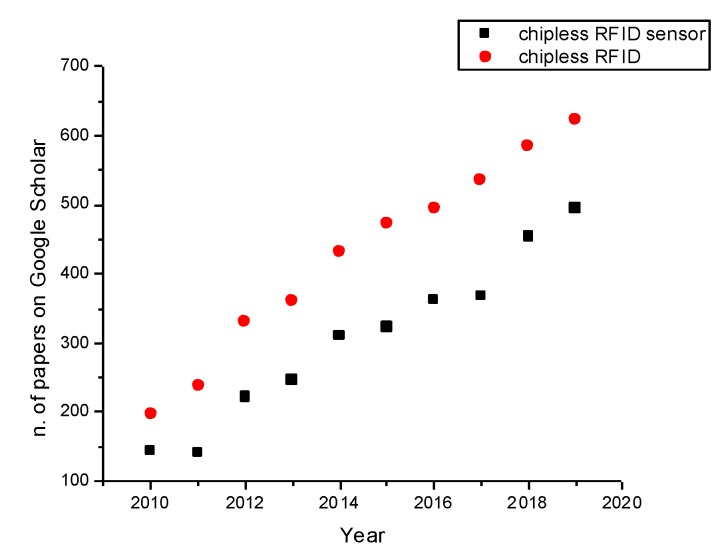
Number of entries per year on Google Scholar using the keywords “chipless RFID” (Radio-frequency identification) and “chipless RFID sensors” as for 24 February 2020.

In Figure 1, the number of entries on Google Scholar corresponding to the keywords “chipless RFID” and “chipless RFID sensor” have been reported as for February 2020. As can be seen, there is a steadily growing interest in the scientific community both in chipless RFID structures and in the chipless RFID-based sensors. More specifically, the interest in the exploitation of these devices as sensors has become the most interesting application field for chipless RFID technologies.

The chipless RFID techniques can be divided into two groups based on the encoding mechanisms: time-domain (TD) [7] or frequency-domain (FD) [8,9]. TD chipless-RFID tags are designed to encode the information in the domain of time. Usually, TD tags are implemented using a propagating structure such as a microstrip and a pulsed interrogating signal that travels along the propagating structure. The information is encoded by using discontinuities at defined positions: the presence of a discontinuity encodes the bit value 1, its absence is the value 0. In order to identify the position of a bit, the delay of the signal must be considered. When an electromagnetic pulse propagates through the substrate, part of it is reflected, in the positions where there is a discontinuity. Such reflections are converted back into electromagnetic energy and retransmitted to the reader. This working principle is called time-domain reflectometry (TDR), and the identifier code ID is represented by the echoes generated by the reflectors. There are different ways to implement time-domain chipless-RFID tags. A compact solution makes use of surface acoustic wave (SAW) resonators that could provide tags with a high number of bits (up to 256 [10]). However, they are far too expensive to be competitive with other RFID solutions, because they require piezoelectric materials. A cheaper and widely used solution exploits the use of discontinuities (or reflectors) located along the main delay line such as a microstrip line. The reflectors can be implemented by introducing bends, stubs or lumped complex impedances placed at defined positions. The reader sends a pulse that propagates through the delay line and is reflected at the points where the discontinuities are placed. The identification code of the tag is constituted by the set of reflections or echoes produced by the discontinuities in the tag. 

The problem with this kind of tags is that the electromagnetic waves propagate along the delay line at the speed of light. In order to produce measurable delays between the echoes, a very short pulse or a very long tag must be used to avoid the overlapping between the different echoes. Very short pulses are quite difficult to obtain in practice and a generator of such pulses is quite expensive. A long transmission line is an alternative solution but the resulting tags are quite large and therefore the bit encoding capability is very limited for tags based on the time domain. 

The FD chipless tag is usually implemented by using multiple resonators tuned at different frequencies. The different frequencies associated with the resonators are distributed within a given frequency band. Consequently, the interrogating signal must have a bandwidth that covers all the resonators frequencies. The effect of a resonator is the production of singularities in the amplitude and/or phase of the frequency response of the tag. In the simplest encoding method, each bit of information is represented by a resonator. The presence of the resonator encodes the bit 1 while its absence represents the bit 0. More sophisticated encoding systems have also been proposed [2,11]. 

In this work, we will focus only on FD chipless tags. The interaction between the interrogating electromagnetic wave and the tag requires a transmitting and a receiving antenna usually in cross-polarized configuration to improve the wireless communication capabilities of the tag, as reported in [8,12]. This kind of FD tags are called retransmission based tags [13]. Another class of frequency domain tags are the so-called backscattered chipless-RFID tags, where the resonant elements provide the information through the spectral signature introduced in the response of the Radar Cross Section (RCS) [2]. The structure of this kind of tags do not require the presence of an antenna, hence the size of the tag is strongly reduced because it contains only the resonators. An example of a very simple backscattered tag is composed of multiple dipoles with a variable-capacity implemented on a dielectric substrate [14,15]. The goal of this technique is to create multiple resonances in the bandwidth of the tag. Similar behaviour can be obtained not only by using dipoles with variable length, but also circular ring resonators or other planar structures. The main limitations of the FD tags are both the bandwidth required to encode a large number of bits and the number of resonant elements that must be increased to increment the information. Consequently, special readers are required and the compactness of the tag is lost when enough information is stored. To reduce or avoid such limitations, several researchers have proposed fractal tags [16,17] or particular geometries [18,19,20] able to concentrate a large number of resonators and, at the same time, keeping their quality factor high.

The limitation in the number of bits is very important in tracking applications, where chipless RFID tags are competing with barcode technology for the labelling of commercial products. The international standard requires a minimum number of bits that has not been met yet by FD tags, and big efforts are currently being made to reach this target.

In sensing applications, the quantity of information stored in the tag is not a critical parameter. While it is true that, in the framework of IoT, it would be desirable to identify the sensing tag with a UID, this has to be made within closed networks and requires much less information than is used in the tracking of goods. Moreover, identification and sensing can be made using similar resonating structures on the same tag. For these reasons, the applications of chipless RFID technologies in the field of sensors are steadily growing in number and variety. 

In this work, we will try to give an overview of the growing amount of information related to the field of chipless RFID sensors, integrating the state-of-the-art for reader architecture and tag structure, presented in Section 2, with a summary of the characteristics and requirements for smart sensing materials, presented in Section 3. In Section 4, tag fabrication techniques are briefly reviewed, while in Section 5 a detailed examination of the most important applications of chipless RFID technology is given. Finally, in Section 6, conclusions and future perspectives are outlined.

## 2. Chipless RFID for IoT Pervasiveness: System Architecture

In this section, the description of two kinds of frequency domain chipless RFID systems is reported. Two different reader structures are presented as well as the structure of different tags equipped with different resonators and coupling structures. The advantages and possible limitations of the different schemas are analyzed and discussed as well as potential applications.

### 2.1. Reader Structure

**Figure 2:**
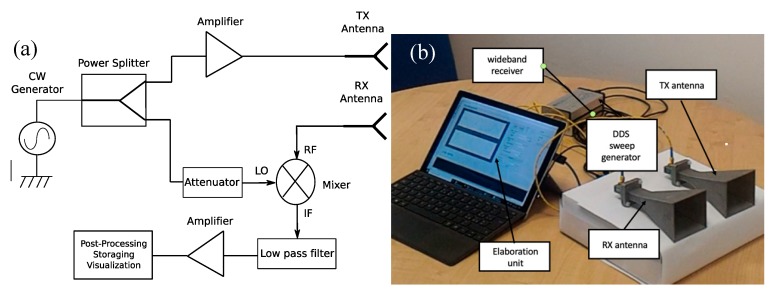
(**a**) Schema of a bistatic reader. (**b**) Photo of a bistatic reader prototype.

In Figure 2a the schema of a classical bi-static reader is reported. It is made of a transmitting and a receiving section. The reader is equipped with two antennas, one is used as a transmitter and the other as a receiver. The transmitting section consists of a continuous wave sinusoidal generator, usually a digital direct synthesizer (DDS) to guarantee a stable frequency signal. The signal provided by the DDS is split using an unequal power splitter and almost all power is sent to a high gain amplifier and then toward the transmitting antenna to generate the interrogating electromagnetic wave. The receiver is composed by a receiving antenna aimed at collecting the reflected wave retransmitted by the tag. The signal is reported in the baseband through a mixer, to retrieve the modulation introduced by the tag. The mixer reference local oscillator is provided by the power splitter. After that, the signal is filtered with a low pass filter, amplified and post-processed [21] with an elaboration unit, usually a microcontroller or a computer. The mixer, lowpass filter, and frequency reference represent a homodyne detector [22,23] able to retrieve the amplitude as well as the phase information. Some reader configurations do not need the homodyne receiver, but it is mandatory if the tag introduces a very low-frequency modulation as in [24,25,26,27,28,29,30,31,32,33,34]. When the tag is equipped with a set of resonators with frequencies near to the frequency of the interrogating electromagnetic wave, the homodyne receiver can be omitted. In this last case, the signal is received, amplified and immediately visualized or post-processed. The only disadvantage of such systems is that they require broadband antennas and the generator must be a sweep generator able to cover all the frequencies covered by the resonators. Such broadband antennas and generators are quite expensive. Another disadvantage is that the number of bits could be limited by the dimensions of the resonators. A real example of a bistatic reader designed to work in the X-band (8–12 GHz) is reported in Figure 2b, where the system makes use of a programmable DDS and a wideband receiver (the devices are from Signal Hund company, Battle Ground WA, USA). The two antennas are two pyramidal horns with a gain of 17 dBi realized in-house.

**Figure 3:**
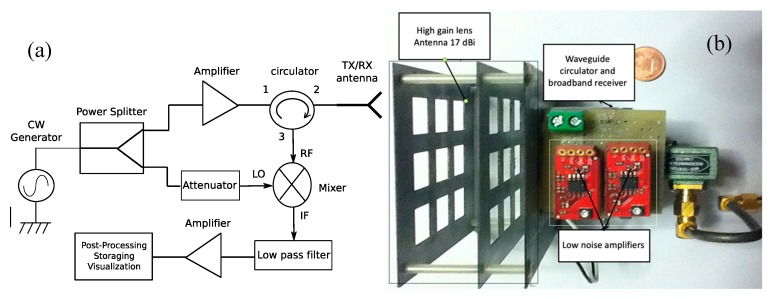
(**a**) Schema of a monostatic reader. (**b**) Photo of a monostatic reader with a waveguide ferrite circulator.

The second kind of reader architecture is reported in Figure 3a, which represents a typical monostatic system schema. In particular, the reader is made with a sweep signal generator, a power splitter, a circulator, a mixer, a low pass filter, and an analogue to digital converter. All the components are similar to those of the bistatic schema, with the only exception of the circulator. This last component is a three ports microwave device that permits the use of only one antenna in both transmitting and receiving mode. The circulator is less expensive compared to another antenna. 

The monostatic reader works as follows: an unequal splitter splits the power produced by the sweep generator, and the lower power is used as a reference for the mixer. The section of the splitter which handles almost all the power produced by the generator is amplified and sent to port 1 of the circulator and is immediately delivered to port 2 connected to the antenna (considered in transmitting mode). Then a signal is reflected by a tag and collected by the antenna which now works in receiving mode. The signal reaches port 2 of the circulator and it is delivered toward port 3 connected to the mixer. As in the bistatic structure, the mixer reports the detected RF signal to baseband, and the low pass filter removes the high-frequency components. The mixer plus, the lowpass filter, is a classical homodyne detector mandatory to receive the signal correctly because the phase information is fundamental to retrieve the information provided by the tag. After the low pass filter, the signal is first amplified and then post-processed and visualized. As can be noticed from Figure 1 and Figure 2 the only difference between the monostatic and bistatic reader is the presence of one or two antennas. Monostatic systems are cheaper because they make use of only one antenna but they are a little bit noisy due to the presence of a circulator.

### 2.2. Tag Structure

**Figure 4:**
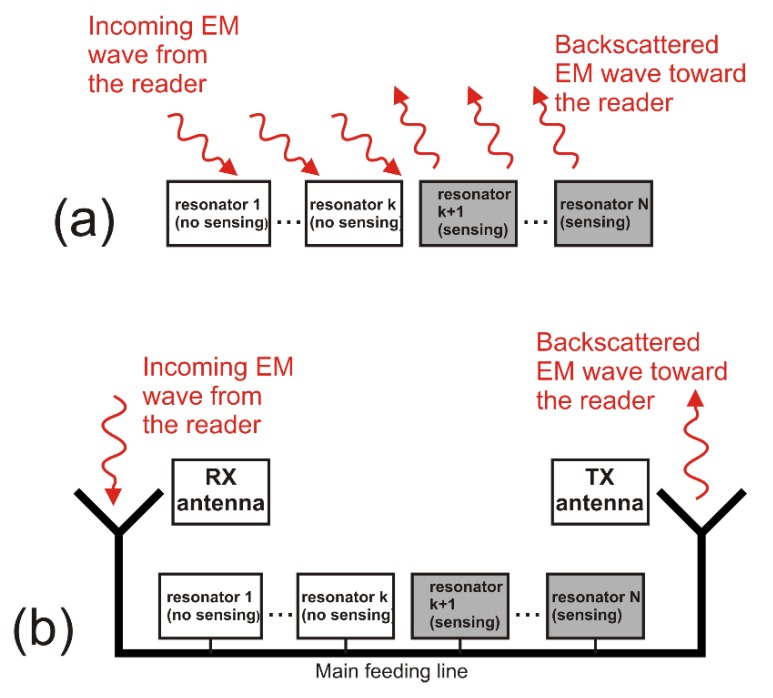
Chipless RFID sensor tag structure including both sensing and coding resonators. (**a**) Backscattered chipless tag. (**b**) Retransmission-based chipless tag.

Backscattered chipless sensor tags have a simple structure because they include only the sensing and coding resonators without feedline and antennas. A scheme of a backscattered chipless tag is reported in Figure 4a, while examples of real backscattered chipless structures are reported in the figures of Section 4. 

The simplest backscattered chipless RFID sensor consists of only a resonator that reflects back to the reader a portion of electromagnetic wave. When the interrogation electromagnetic wave generated by the reader impinges on the tag, it interacts with the resonator generating induction currents which reflect back towards the reader a portion of the RF signal. The amount of backscattered energy depends on the efficiency of the resonator. 

Usually, these kind of chipless RFID tags include a set of resonators and the interrogating electromagnetic wave is not a single tone continuous wave but a broadband signal. The resonators produce negative peaks at particular frequencies of the spectrum, encoding the data. The sensing resonators differ from the coding resonators because the peak position depends on some environmental variable. The main drawback of such devices is the limited range at which they can operate, which is reduced to a few centimetres. The general structure of retransmission-based FD chipless sensors is shown in Figure 4b and usually comprises a set of resonators in cascade form, the main coupling line, and two antennas. 

Circular polarization is usually preferred because it is insensitive to the reader position [35,36]. When the RF signal impinges on the tag receiving (Rx) antenna, is converted into RF currents and propagates further towards the resonating circuit. A series of resonators are aimed at producing phase jumps at particular frequencies of the spectrum. The resonators encode the data bit or the sensing information and are coupled with the main feeding line employing capacitive coupling. Then, when the signal has been passed through all the resonators, the unique spectral signature of the tag encoding the information is transmitted back toward the reader employing the tag transmitter antenna (Tx). 

**Figure 5:**
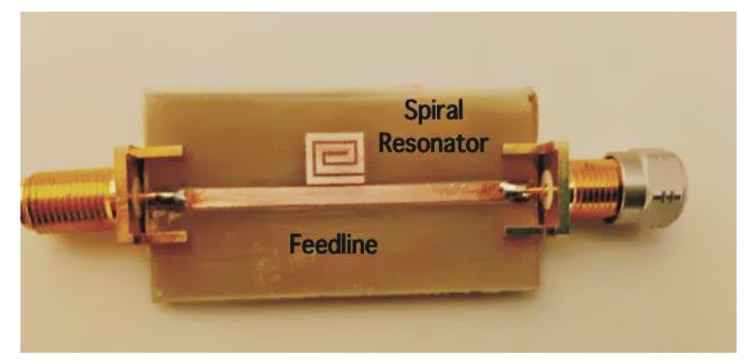
Photo of a single-bit tag structure.

**Figure 6:**
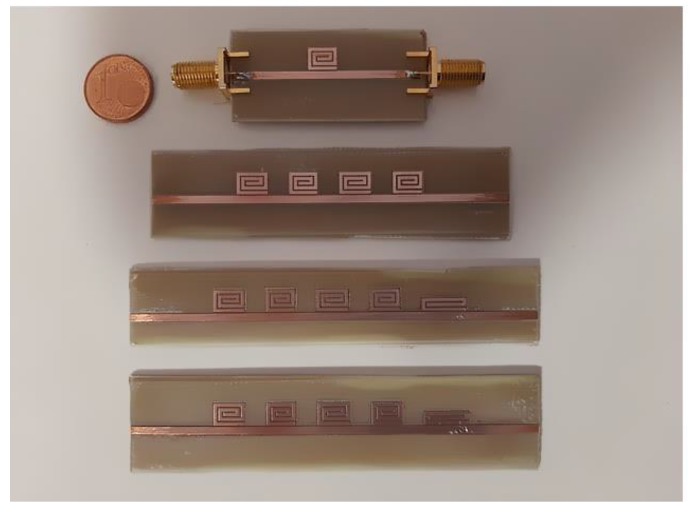
Photo of chipless tags equipped with different numbers of spiral resonators.

**Figure 7:**
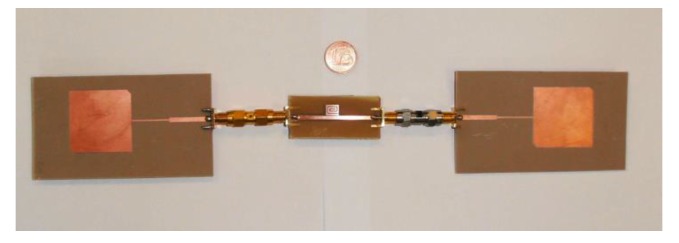
Photo of a single-bit chipless tag equipped with two patch antennas with modified corners.

A prototype of a single-bit retransmission-based chipless tag is shown in Figure 5. As can be noticed, the mainline is a microstrip and the bit is encoded with a spiral resonator. Figure 6 shows several chipless tags equipped with a different number of resonators. To receive and retransmit the electromagnetic wave the tag is usually connected to two antennas as reported in Figure 7, where the single-bit tag shown in Figure 5 is connected with two circular polarized antennas using subminiature type A (SMA) coaxial connectors. The circular polarization has been obtained cutting the corners of two rectangular patch antennas tuned at a frequency of 2.45 GHz.

**Figure 8:**
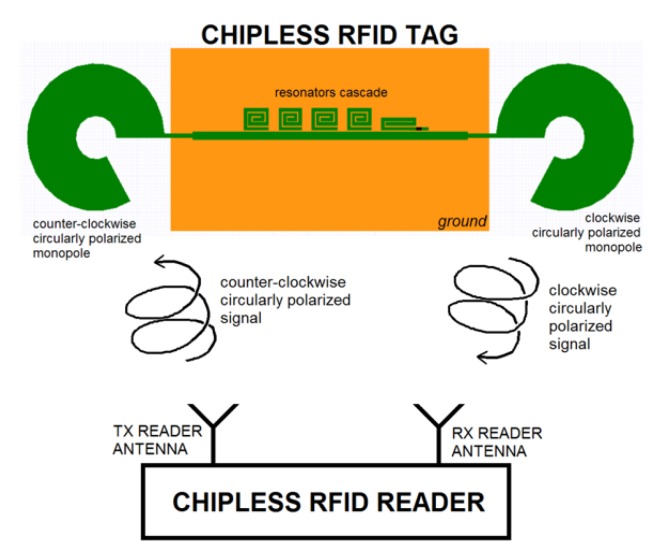
Schema of a chipless tag system operating in circular polarization.

**Figure 9:**
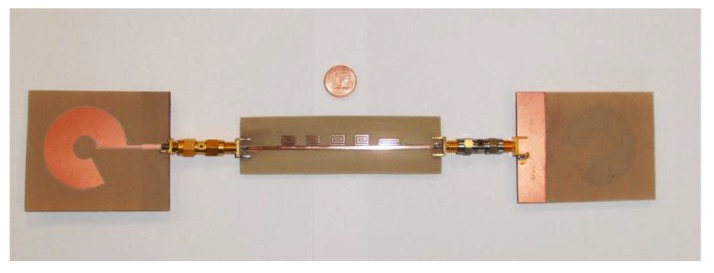
Photograph of a five-bit chipless tag equipped with two broadband circular polarized antennas.

As stated above, to make the chipless tags insensitive to position, circular polarized antennas are used instead of linear polarized antennas as depicted in Figure 8. A photo of a chipless tag able to encode five bits and equipped with two broadband circular polarized antennas is reported in Figure 9.

The sensing ability to environmental parameters can be obtained covering one of the resonators with an appropriate sensing material, as will be discussed in more detail in Section 3. This last strategy is easier to implement in case of backscattered chipless sensor tags, that are smaller, have a simpler structure and are easier to integrate on non-planar surfaces. 

**Figure 10:**
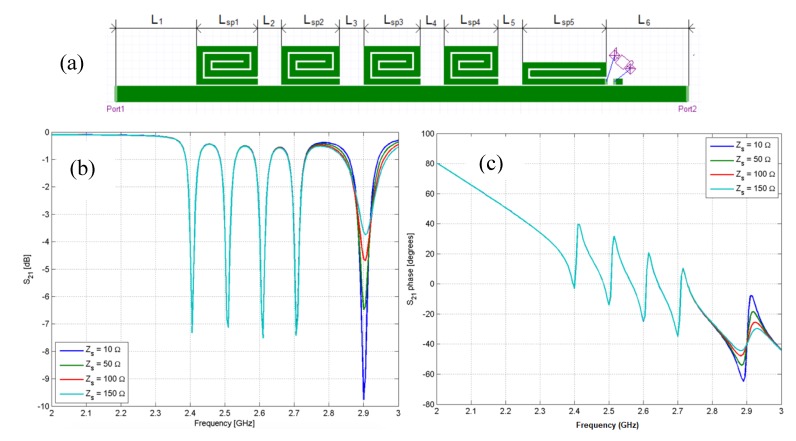
(**a**) Schema of a five-bit chipless tag equipped with a sensing element placed on the fifth resonator and a simulated five-bit chipless tag equipped with different resistors placed on the fifth resonator. (**b**) |S_21_| and (**c**) phase response.

A chipless tag can also be used as a sensor by adding elements, such as a thermistor or a variable capacitor, able to measure environmental parameters like temperature or humidity. The additional elements can be directly connected to a resonator as reported in Figure 10a, where a sensing element is mounted on the last spiral resonator. 

Figure 10 reports the simulated response of the S_21_ versus the frequency in magnitude (Figure 10b) and phase (Figure 10c). The peaks due to the presence of resonators are clearly visible and the change of resistance connected to the last resonator produces an evident effect on the resonance of the fifth resonator. 

**Figure 11:**
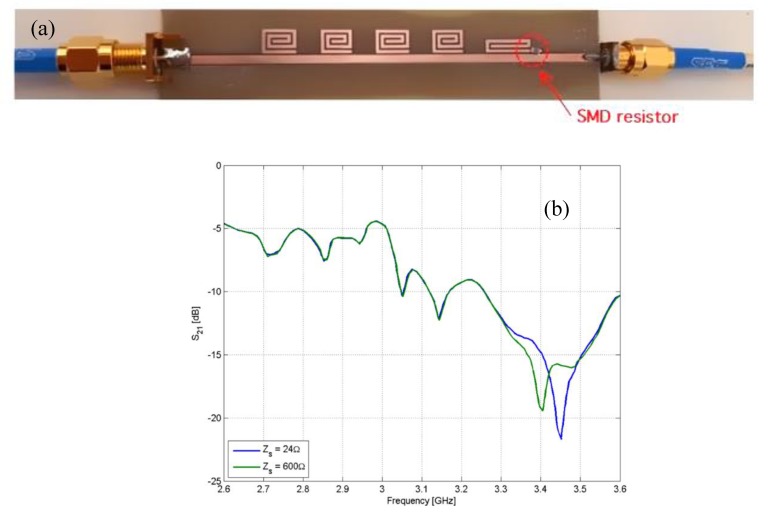
(**a**) Photo of a five-bit chipless tag equipped with a thermistor and (**b**) measured |S_21_| response of a five-bit chipless tag equipped with a thermistor.

Figure 11a reports a photo of a prototype where a thermistor has been mounted on the last resonator to obtain a chipless temperature sensor. The measured response of the prototype is reported in Figure 11b. As can be noticed, the change of resistance not only produces a change in the peak amplitude but also a frequency shift which is likely due to the parasitic inductance and capacitance of the sensing element.

A common issue related to chipless RFID sensing is the reading range, which is usually limited compared to traditional RFID technologies [37]. This is especially true for backscattered chipless tags, where the environment electromagnetic noise strongly limits the maximum reading distance. 

**Figure 12:**
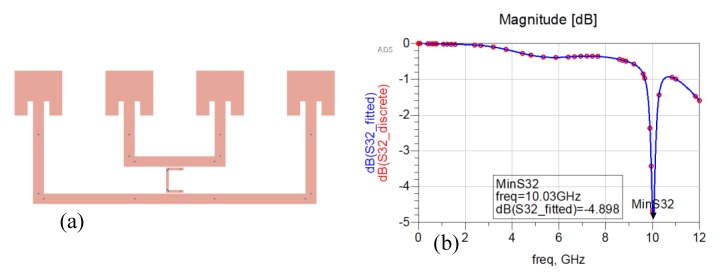
(**a**) Single-bit chipless linear Van Atta array composed by four elements with a single C resonator. (**b**) Simulated backscattered response in the X band (8GHz–12GHz).

An interesting way to develop a chipless RFID tag with an increased reading range makes use of a Van Atta array [38]. A Van Atta array is an antenna that reproduces the effects of a corner reflector through a planar configuration and is commonly used as radar reflectors [39]. This behaviour is obtained because of the particular arrangement of its radiating elements. The goal of a Van Atta array is to collect the energy of an impinging planar wave and to retransmit it in the same direction of arrival (DoA). Since we are dealing with concepts related to planar waves that exploit far-field principles in their communications, this means that long-range chipless RFIDs can be developed. The design of a single-bit chipless RFID based on a four-element Van Atta array is reported in Figure 12a. It is composed of four inset rectangular antenna patches, tuned at the central X microwave frequency band (10 GHz) and linearly arranged, with the interelement distance equal to a quarter-wavelength at 10 GHz. The antenna insets have been designed to match the antenna impedance at 50 Ω. Couples of antenna elements are connected through microstrip lines with a characteristic impedance of 50 Ω. The length of the delay lines interconnecting opposite elements has a strong impact on the correct direction of back radiation. Linear Van Atta arrays can receive the planar excitation from any DoA in an angular range (0°, 180°) and they are able to retransmit it in the right direction. More detail on Van Atta array design can be found in refs. [40,41,42,43]. To encode bits of information, one or more resonators can be placed between the microstrip connections lines. In Figure 12a a single C shape resonator is placed between the connection microstrip lines of the four-element Van Atta array and represents a bit of information. The simulated response measured at the reader is reported in Figure 12b. The resonator is tuned at the central frequency of the X band and its presence is evident in the simulated backscattered wave. 

**Figure 13:**
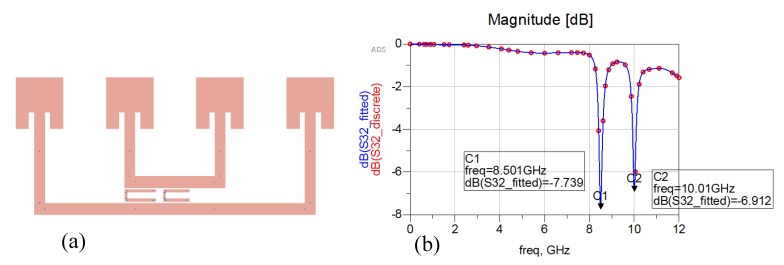
(**a**) Two-bit chipless linear Van Atta array composed of four elements with two C resonators. (**b**) Simulated backscattered response in the X band (8GHz–12GHz).

**Figure 14:**
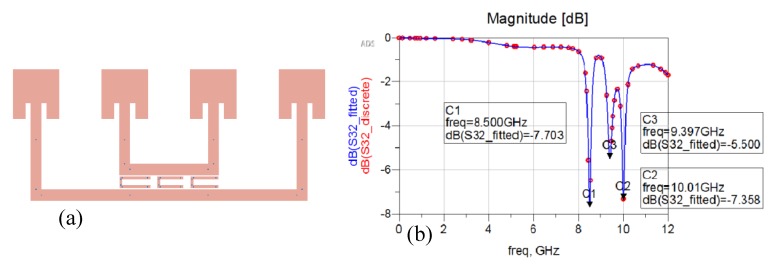
(**a**) Three-bit chipless linear Van Atta array composed of four elements with three C resonators. (**b**) Simulated backscattered response in the X band (8GHz–12GHz).

Figure 13a and Figure 14a show a two- and three-bit chipless RFID tags based on the same four-element Van Atta array. They have been obtained by inserting two and three C shaped resonators between the microstrip connection lines. Figure 13b and Figure 14b report the simulated response of the two-bit and three-bit tags, respectively. As can be seen, in both cases the information carried by the backscattered wave is quite clear and strong.

**Figure 15:**
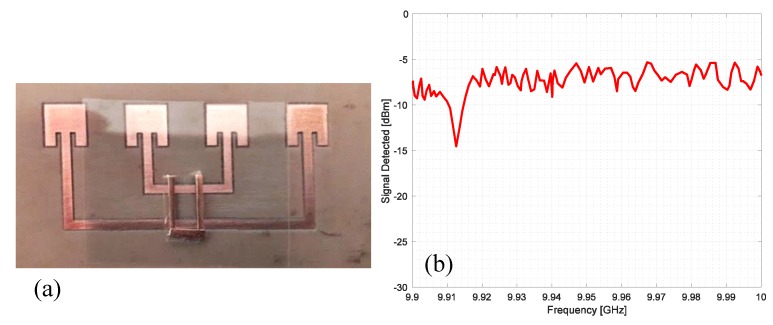
(**a**) Photograph of a single-bit chipless Van Atta array RFID prototype fabricated on an FR4 dielectric substrate and equipped with a C resonator placed vertically with respect to the connection microstrip. (**b**) Measured signal from the prototype reported in (**a**).

An alternative but similar way to insert information is to place the resonators aimed at encoding the information directly on top of the Van Atta array. To keep the array behaviour unaltered, a thin sheet of plastic material must be positioned near the microstrip connection lines to separate the array from the resonator, as shown in the prototype in Figure 15a. The Van Atta array has been fabricated with a computer-numerical-control milling machine on FR4 dielectric substrate (thickness 1.6 mm, ε_r_ = 4.48, tan(δ) = 0.01). A C shaped resonator, fabricated with copper tape and tuned at 9.15 GHz, is placed vertically along the Van Atta array connection lines.

The measured backscattered wave is reported in Figure 15b, where the negative peak generated by the C shape resonator at 9.15 GHz is clearly visible. The power of the generator of the interrogating electromagnetic wave was 10 mW, whilst the transmitting antenna was a pyramidal horn characterized with a gain of 17 dBi. In this and all the next experiments, the distance between the reader and tags was 5 m.

**Figure 16:**
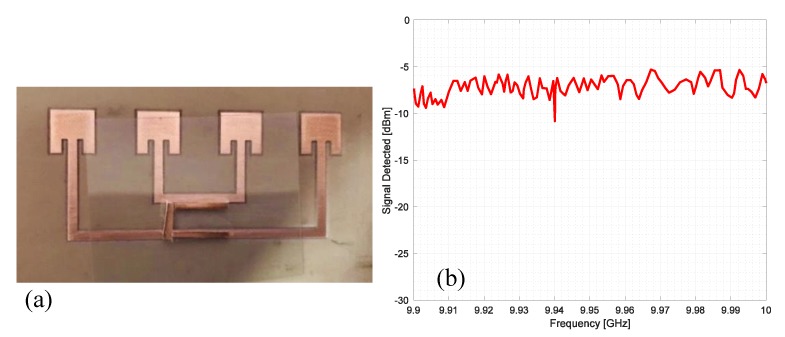
(**a**) Photo of a single-bit chipless Van Atta array RFID prototype fabricated on an FR4 dielectric substrate and equipped with a C resonator placed horizontally with respect to the connection microstrip. (**b**) Measured signal from the prototype reported in (**a**).

To analyze the effect of the resonator orientation, the same resonator has been placed horizontally with respect to the connection lines as reported in Figure 16a. The corresponding measured backscattered signal is reported in Figure 16b. In this case, the presence of the resonator is not noticeable and the peak that should be located at 9.15 GHz is completely absent.

**Figure 17:**
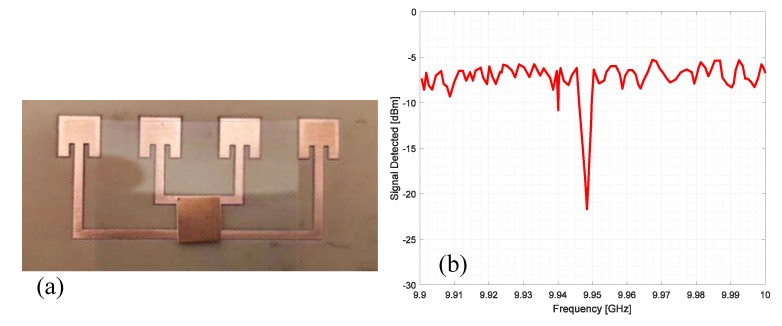
(**a**) Photo of a single-bit chipless Van Atta array RFID prototype fabricated on an FR4 dielectric substrate and equipped with a square patch resonator. (**b**) Measured signal from the prototype reported in (**a**).

In the last experiment, a square patch antenna has been considered as a resonator, and the dimensions were chosen to obtain a peak at about 9.95 GHz. The photograph of the prototype is reported in Figure 17a. The square resonator has been placed exactly in the mechanical boresight of the array to obtain a good coupling with the microstrip line. The measured signal is reported in Figure 17b. In this case, the peak is quite strong and clear and the square resonator works better than the other considered resonator. In fact, the peak of the square resonator reaches a deep of about −22 dBm to the reference level, while the C shape resonator shows a depth of about −15 dBm.

## 3. Smart Materials for Chipless Sensing

In the previous section, the discussion was concentrated on the structure of the chipless tag and its general suitability as a sensor for IoT needs and requirements. In this section, we focus on the more specific part of the sensor, which must be tailored to the single variable to be measured and, ultimately, determines the selectivity and sensitivity of the sensor itself. This important part is the material used for the detection of a specific environmental variable and should possess some unique features [44]. 

It should be noted that chipless sensors without a dedicated sensing material have been widely reported in the literature [45]. These sensors exploit other characteristics intrinsic to the chipless structure, such as the variation of the physical or electrical length of the resonator, or the change of RF coupling between a resonator and a microstructure. They have the advantage of structural simplicity but are rather limited in their applications. Among them can be listed proximity, rotation or movement sensors, as well as structural health and stress monitoring sensors [46,47,48].

In a more general view and in order to broaden the number of applications, the sensing material is of paramount importance for a well-performing sensor. While an enormous amount of literature has been published on chipless RFID structural capabilities from the architectural, telemetric, and storage potentiality point of view, only a limited part of the literature focuses on the sensing material properties and functionality.

The reason for this imbalance is not unique. Chipless sensors are a relatively new field and are a domain where telecommunication engineering has to meet material science, merging different expertise that may take some time to blend appropriately. Moreover, in chipless RFID sensing, dielectric properties of materials at microwave frequencies are the key to an appropriate selection, but these properties are seldom considered in sensing technologies, that usually exploit other parameters like variation of resistance, capacitance or optoelectronic properties.

A good sensing material for a chipless sensor is a material that changes its dielectric properties when exposed to the specific physical or chemical variable to be sensed. In this regard, the chipless sensor features an important advantage, because the dielectric parameters that may vary are both the real and the imaginary part of the dielectric function, that is, both the frequency and the Q factor of the resonating structure. The relation between frequency and Q factor of the resonator and dielectric function of the medium surrounding it is neither linear nor easy to calculate. It depends on several variables including the resonating geometry and metal conductivity, the operating frequency, the substrate material and, of course, the sensing material characteristics, including its thickness and geometry. A good prediction requires computer-based simulations and therefore necessitates not only appropriate simulation tools but also the knowledge of geometrical and material characteristics.

However, a more empirical approach may be used, calibrating the sensor at different values of the monitored variable, as is typically made with other typologies of sensors. To verify the suitability of existing materials, or, alternatively, to produce a material tailored for the detection of a defined variable, it is necessary to understand which are the factors affecting the dielectric characteristic of a material and their changes.

The dielectric properties of materials [49] are characterized by the complex permittivity ε*, which has real and imaginary components ε and ε′ and by the loss tangent, or dissipation factor, defined as tan(δ) =ε′/ε. The dielectric properties are controlled by and can be broadly predicted from their chemical structure. Especially for plastic materials, their polar or non-polar nature can be explained by the chemical composition and this essentially determines the material electrical performances.

Certain molecules and groups display higher polarizability than others. Aromatic rings, sulfur, iodine and bromine are highly polarizable, and the presence of these groups increases the dielectric constant. Conversely, fluorine which has a small atomic radius and a concentrated negative charge has low polarizability and will decrease the dielectric constant of the material containing it.

Most microwave materials are formulated for low dispersion and minimal change of tan(δ) with frequency or temperature or environmental parameters but a good sensing material must have very different characteristics. 

This considerable variation of properties in the microwave region is normally considered a nuisance in the design of microwave components but may offer new great opportunities in sensing technology. In this spectral region, a small variation of material composition due to an environmental change can be converted in a high variation of dielectric properties, paving the way for sensors with high sensitivity and selectivity exploiting this frequency band. 

Finally, most sensing materials are not perfect dielectrics and therefore may show a limited, but not negligible, conductivity. This has important consequences because even low values of conductivity can greatly increase the losses in the material and the dissipation factor. Practically speaking, this means that a resonator surrounded by the weakly conductive material will show a low Q factor, but on the other hand, conductivity changes due to environmental variables can be detected measuring the Q-factor of the chipless resonator. Many well-known sensing materials exploit conductivity changes (carbon nanotubes, metal oxides, silicon nanowires, organic semiconductors) and this relation opens the way to their exploitation in chipless RFID sensing. 

Unfortunately, as chipless sensing is a new field of study, information on the dielectric properties of sensing materials at microwave frequencies and their variation with environmental variables is rather scarce in the literaure, and it is very likely that the basic knowdlegde in this field will increase rapidly in the future. A few examples of sensing materials and their dielectric properties are reported in Table 1. More specific information and detailed examples are described in Section 5.

## 4. Sensor Fabrication Techniques

Chipless RFID tags can be fabricated by simpler and cheaper processes than those used for conventional RFID tags. This fundamental difference is due to the absence of the silicon chip and therefore there is no need for a manufacturing process suitable for microelectronics. In addition, there is also no need to connect the antenna to the chip [2]. Practically speaking, this means that a chipless tag can be more robust and much less expensive than a tag with a chip. The sensing capability can, however, add some complexity to the tag fabrication, depending on the materials used for sensing, if any, and on its sensing geometry. It is clear that, in view of wide exploitation within IoT, chipless tag structures need to be produced with extremely low-cost manufacturing processes, and, thanks to the recent and fast development of printed electronics, this possibility is now a reality.

While discussing the most important sensor fabrication techniques, it is important to distinguish between the fabrication of the resonating structure and production of the specific sensing material. The two parts of the sensors are usually produced with different technologies, and their integration may require special care in designing the whole fabrication flow. Moreover, the type of application and the sensing methodology may use non-standard microwave materials, with associated non-standard production methods. As an example, flexible and conformable tags must be produced on a polymeric material substrate, but the conductive and the sensing material must have the same flexibility. Plastics substrates have often a low softening temperature and limited chemical resistance, and this can prevent the use of specific deposition techniques for the sensing part. Furthermore, the conductive resonating structure must be realized on metal or in a very conductive ink of appropriate thickness, limiting the choice of the materials available. In conclusion, the main fabrication limitations have to be faced and overcome on a case-by-case basis, and the available solutions have to be found keeping in mind both materials and design constraints.

A summary of the most relevant techniques employed to fabricate chipless RFID sensor tags is reported in Table 2. Fabrication techniques are then discussed in more detail in the following part of this section.

### 4.1. Micro and Nanolithography

Microlithography is the traditional technique used for micro- and nano-devices fabrication, including electronic industry and PCB (printed circuit board) circuitry. It includes several separate techniques but is fundamentally based on the repetition of a three-step process: deposition, lithography and etching. 

While the electronic industry typically uses silicon wafers as substrates, PCB manufacturers can provide substrate plates with laminated and bonded copper layers on one or two sides. Other substrates, like plastic or fabric, can be used as substrates, provided they are conveniently metallized or covered with a layer of conductive material. This metallization process is necessary to create the resonators, the antennas and the microstrip lines that must be realized in a highly conductive material. The photolithography process is then performed covering the surface of the tag with photosensitive resin and the pattern transfer is then realized exposing the tag to ultraviolet light through a mask that contains the geometric patterns. When the unwanted parts of the resist layer have been removed, etching is performed to define the metal. A similar process can be used to apply the sensing material on top of the resonators.

**Figure 18:**
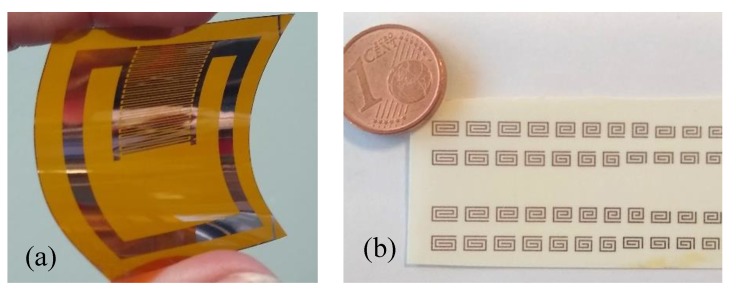
Tags realized by lithography. (**a**) Flexible sensing resonator structure (copper on Kapton). (**b**) Spiral resonator arrays (copper on 168 μm-thick Rogers 4350B).

Unlike traditional microfabrication, which is usually performed on layers of sub-micron thickness, PCB-type substrates have a metal thickness of several microns (typically 17 or 35 μm), which limits accordingly the achievable resolution. Even in case of metal structures fabricated by sputtering, electrodeposition or other metallization techniques, where the thickness can be tuned to the needs of the final device, microwave resonators must have a minimum thickness around 1–2 μm, depending on their frequency range and metal conductivity. Commercial antennas for chipped RFIDs in the ultra-high frequency range are typically fabricated in aluminium by lithography, even on flexible substrates. In this case, the resolution constraints are not a problem due to the device dimensions, and the fabrication is relatively cheap, especially in the huge numbers typical of mass production. Examples of resonator structures realized by microlithography are reported in Figure 18.

The application of the sensing material can be obtained with a similar photolithographic process, even though there are many other possible methods. The required resolution for the sensing material is rather low, and less precise methods, like drop-casting or thermo-compressive bonding, are suitable for applying the materials.

### 4.2. Screen and Inkjet Printing

Screen printing is the most common industrial technique for conductive material deposition on flexible substrates. This technique involves the deposition of conductive polymer films on plastic or fabric using a stencil technique to define the desired pattern. The ink is pressed onto the substrate through a screen with a blade, while the stencil mask protects the areas where the ink should not be deposited. The thickness of the conductive film can be increased with multiple printing cycles. This technique can be coupled to roll-to-roll techniques, proving a very high throughput and is therefore very suitable for tag mass production [59].

Screen printing has been used in RFID technology for printing both antennas [60] for wearable applications and chipless sensor tags [61], with good results. The minimum feature size is however limited to 50 to 100 μm by the stencil approach and by fabric substrate inhomogeneities. This limitation becomes important as wireless connections move towards higher frequency bands, as is foreseen in the future, requiring smaller antennas and components.

Inkjet printing is a more versatile technique so far mostly employed for small productions or research purposes. It is considered one of the emerging additive manufacturing techniques and can be used for depositing patterned layers of a large variety of inks on several types of different substrates, including paper, plastic, special polymers and fabric, with a minimum feature size that can reach 20 μm or even less with new related printing approaches like aerosol printing. In RFID technology inkjet printing has been used for the realization of RF components on a wide range of substrates using conductive inks [62,63]. In the chipless RFID field, the exploitation of this technique is essential for manufacture tags with high performance and with the quality required for fully IoT integration. 

**Figure 19:**
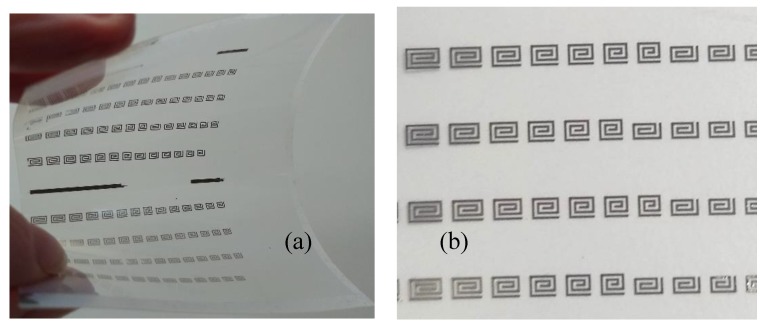
(**a**) Ink-jet printed resonators arrays on polyethylene terephthalate substrate realized with highly conductive Ag-nanoparticles ink. (**b**) Detail of the same sample.

Compared to other printed electronic components, chipless RFIDs have requirements that are more severe on the ink characteristics. Highest conductivity inks are based on silver nanoparticles and can have conductivities up to 10^7^ S/m, which is only one order of magnitude less than copper. These performances are adequate for applications in the microwave range, but other conductive materials widely used in printed and organic electronics, like graphene-based inks or organic conducting polymers do not meet the minimum conductivity requirements for this application. On the other hand, the versatility of inkjet printing allows for deposition of both conductive and sensing material using two different inks on the same substrate, avoiding problems of alignment between different layers that are typical of microlithography and most integration techniques. An example of resonator arrays realized by ink-jet printing is reported in Figure 19.

### 4.3. Other Fabrication Techniques

Large resonators on rigid structures can be realized with common milling techniques. This approach is rather slow and has limited resolution but it is useful for prototyping. In addition, it can be coupled with dedicated deposition techniques for smart sensing materials when this is needed. The sensing material can be applied on top of the resonators by drop-casting, spin coating or, thanks to the rigidity of the resonator substrate, even by thermo-compression bonding.

Special applications may require unusual fabrication techniques. Wearables embedded in garments are one of these, and, giving the large dimensions of some wearable RF components, techniques like e-thread based embroidery have been reported both for [64,65] antenna and chipless RFID tag [66] fabrication. E-threads are made of metal-coated polymeric filaments that are stitched upon a fabric substrate manually or automatically, enabling the realization of conductive traces. This technique can be applied to a variety of substrates, but, as it is easily understandable, has a very limited resolution and can therefore only be used for large low-frequency antennas. Other simple and low-cost techniques have been reported, such as the fill until full [67] technique, that can also be used for low-resolution patterns.

## 5. Applications

### 5.1. Physical Sensors

Several approaches have been proposed to realize a chipless RFID tag with an integrated sensor [45], and the choice among the different options depends mainly on the specific parameter to be sensed. Physical parameters like temperature and humidity were among the first examples reported in the literature. 

In fact, sensors capable of remotely measuring high temperatures in harsh environments are needed for a variety of applications. For instance, they are very helpful to prevent structural failure due to high temperatures, or for high-temperature monitoring of heat resistant materials, or again for high-temperature testing in engines or qualification testing of disc brakes. Chipless sensors, having no electronics in the tag, have the potentialities to address most of these challenges [68,69]. Temperature sensors are needed also for environmental monitoring, and wireless low-cost ubiquitous temperature sensors are desperately needed for the future IoT. Temperature chipless sensors are well represented in the literature [70,71,72,73,74,75,76]. Very sensitive solutions have also been reported but at the cost of losing printability and introducing structured multilayer fabrication [77,78,79,80]. 

Humidity is one of the most important physical parameters for assessment of air quality in indoor and outdoor environments, in agriculture optimization, in food conservation monitoring and in water damage assessment in buildings. Several smart materials have been identified for chipless humidity sensor tags, from Kapton HN [81] to Polyvinyl-alcohol (PVA) [82], silica-gel [83] and silicon nanowires [58,84,85]. Tags that enable both humidity and temperature monitoring [61,71,86,87] have also been proposed as well as solutions that combine identity tagging with sensing [85]. Unconventional detection schemes using metamaterial approaches are also reported [86]. 

Chipless pressure sensors are also present in the literature, both SAW-based [71] and frequency-based [86]. More complicated and expensive fabrication schemes can make the frequency-based solutions suitable for very high temperatures [88].

### 5.2. Chemical Sensors

Chemical sensors are more complex to implement in a chipless sensing structure, and in addition, they face a common issue typical of all type of chemical sensors, which is the selectivity towards a specific analyte over others. Nonetheless, chipless tags for gas chemical sensing have a commercial interest [89] and have been reported mainly exploiting carbon nanotubes as smart material [54,90]. These tags, produced by inkjet printing, have been demonstrated to be able to monitor both ammonia and NOx gases for environmental monitoring, as well as CO_2_ [53] and temperature [55]. An easy-to-fabricate sensor for ethanol gas detection is reported in [91]. Other, more complicated passive sensors schemes have been reported for ethylene [92] monitoring, aimed at detecting fruit ripening [93]. 

Chemical composition analysis of liquids employing chipless resonating structures is well represented in the literature [57,94,95,96,97], often coupled to microfluidic structures [98,99,100,101]. In fact, all the approaches reported for analysis of liquid solutions exploit the difference in dielectric constant caused by a different solution composition, detectable through a shift of the resonance peak. Since water has, by far, the highest dielectric constant and the highest loss factor compared to other common liquids, it is normally used as one of the components. In this respect, interesting applications are foreseen in water–oil interface detection in the treatment of oil sands [57].

### 5.3. Smart Packaging

RFID technologies have found many applications in automatic tracking and identification of objects and food-related goods. While chip-based solutions provide high-coding capacity and can easily comply with international product code standards, their cost makes them expensive when compared to the more widespread barcodes and therefore a lot of research effort has been made to produce chipless tags with high coding capacity [3,102,103]. However, a smart package can have more functionalities than just tracking items, because sensing capabilities can be added, including parameters that can give information on the item storage environment, age or authenticity.

Intrinsic properties of perishable and food products may change after packaging and this can lead to a loss of quality [104]. Depending on the contents of the package, biological, chemical, or physical processes occur, which ultimately lead to degeneration of the product, but these changes are in most cases difficult to assess by the consumers.

Smart and intelligent packaging requires solutions exploiting simple manufacturing techniques making use of low-cost and high-throughput printing technologies. Chipless tags are clearly [105] an excellent opportunity for this commercial sector, because of their cheap production cost and printability [59].

Packages are usually made with some paper substrate, and paper substrates have been considered as a low-cost and environmental-friendly [106] alternative to plastic substrates for printed electronics. Compared with photo paper, commercial packaging paper is considerably cheaper and more robust in a harsh environment, and therefore, directly using the packaging paper as printing substrate [105] can reduce both the manufacturing steps and cost. In addition, packaging paper, as it is or properly functionalized, can be used also as sensing material [107], easily merging the fabrication process of the sensor tag into the conventional package production flow.

Packaging paper presents two important drawbacks for chipless tag printing when compared to traditional substrates or plastic: high roughness and high substrate losses. Roughness strongly influences ink conductivity [108] but can be reduced by overprinting [105], with good results, especially at low frequencies. On the other end, losses have also a more important impact as the frequency increases and this is likely the reason why most chipless tags reported in the literature for smart packaging work at low frequencies [105,109]. Chipless sensors on a package, printed directly on paper with conductive ink, are usually humidity sensors [107,110], often coupled with identification tags on the same package. This is because dielectric properties of paper and cardboard are humidity dependent, making it a suitable material both for the tag substrate and the sensing material. For the monitoring of other parameters, such as pH or temperature, additional materials need to be included in the sensor [111] to be embedded in the package, at least to isolate the identification resonators from the sensing ones [110] or to differentiate the resonators in multiparametric sensing [55,111].

Intelligent packaging applications are expected to grow tremendously in the near future, in order to improve both the tracking and the quality of the stored and transported goods in the framework of IoT and in preserving the quality of transported food. Chipless RFIDs are a very promising technology [112] for these applications, and the currently demonstrated prototypes are paving the way for more complex, sophisticated and unexpected applications. As an example, applications in the tracking of pharmaceutical products have been proposed [113], where the chipless tag is included in the pills to address the severe issue of drug counterfeiting that may sometimes become fatal. Very specific sensors for food pathogens have also been proposed, coupled with a microfluidic structure [114]. 

### 5.4. Structural Health

The high costs associated with potential failures have made structural health monitoring an urgent and necessary security measure to ensure operational safety of large-scale structures and machinery such as railways, pipelines, bridges, and buildings. The implemented strategies concerning the safety of persons and goods in such structures are using an increasing number of wireless sensing technologies. By eliminating electric wiring, wireless sensor networks can distribute sensors over a large area and with high density, solving many complications related to structural monitoring. However, current wireless sensing applications generally make use of battery-powered sensors, which are quite expensive and have limited battery life.

Several approaches using less costly passive RFID networks have been proposed [115,116,117,118] but chipless RFID tags can dramatically lower the cost of RFID technology, being completely printable and potentially mass fabricated using low-cost conductive materials. Moreover, they are capable to work in harsh environments where electronic devices cannot survive. Sensing chipless structures can thus be mounted or embedded on critical structures as a smart-skin [119].

The defect information can be related to a resonant frequency shift [120] or to variations of other electrical parameters. Mechanical defects can be detected, but also cracks and corrosion phenomena. When applied to buildings or large structures, these approaches require a large reading range, which can be an important limit in their practical application, and special solutions are required [119,121], as mentioned in Section 2.

Many recent works on strain and crack sensors using RFID and chipless technology are present in the literature, using both conventional manufacturing techniques [122] or inkjet printing approaches [123]. 

However, many of the sensing approaches proposed do not require any smart material, but only a purposefully designed antenna [124,125]. Deformation and strain change the electrical length and therefore the resonant frequency of the antenna. Cracks are detected in similar ways [126,127,128,129] while adequate design and data elaboration can discriminate the crack orientation [130]. Strain can also be monitored using patch antennas loaded with open-loop stubs. This approach can determine also the strain direction [131]. In order to reach an adequate strain resolution in a high dynamic range, an adequate antenna material with a large structural change in response to a small applied strain is needed, and this is the critical point of this simple technology. 

Chipless RFID sensors can also monitor corrosion of metallic structures. During the initial phase of a corrosion phenomenon, a thin layer of oxide appears on the metal surface, and this causes changes in the conductivity and permittivity of the metal. These changes depend on the metal type and can be monitored by the impedance change of tag antennas [132]. Alternatively, corrosion can be prevented before it takes place detecting air or water infiltration exploiting the resonator frequency shift caused by the change of local permittivity [133,134,135]. Multiparametric approaches to corrosion are also reported [136].

### 5.5. Position, Displacement and Touch Sensors

Accurate indoor localization has recently gained great interest for a variety of future location-based services, and passive RFID technologies are the most obvious choice for this purpose. In fact, smart and flexible localization exploiting wireless technologies is one of the pillars of future IoT development and growth.

Chipless RFID technology, with no battery and no chip, is an extremely low-cost solution and is applicable for mass deployment in a large variety of localization applications. For this reason, localization using chipless tags has extensively been investigated in the literature.

Most of the papers on this topic are focused on the elaboration algorithms and measurements techniques and are less concerned about the tag structure. For these applications, the focus is clearly more on the reading strategy than on the tag architecture and material, which are limited to the choice of the most appropriate resonating structure to be coupled with a suitable identification scheme.

Many approaches to chipless tag localization use time-resolved techniques [137,138,139] typical of widespread chipped RFID technologies, such as round-trip time of flight [140], or phase acquisition [141,142]. Frequency coded approaches are also present [143,144,145], even though detecting and localizing the chipless tags could be challenging because the indoor signal propagation environments are complex and the backscattered signal from the tag is weak [146,147,148]. To overcome these limitations, suitable radar techniques have been developed [119,149]. 

**Figure 20:**
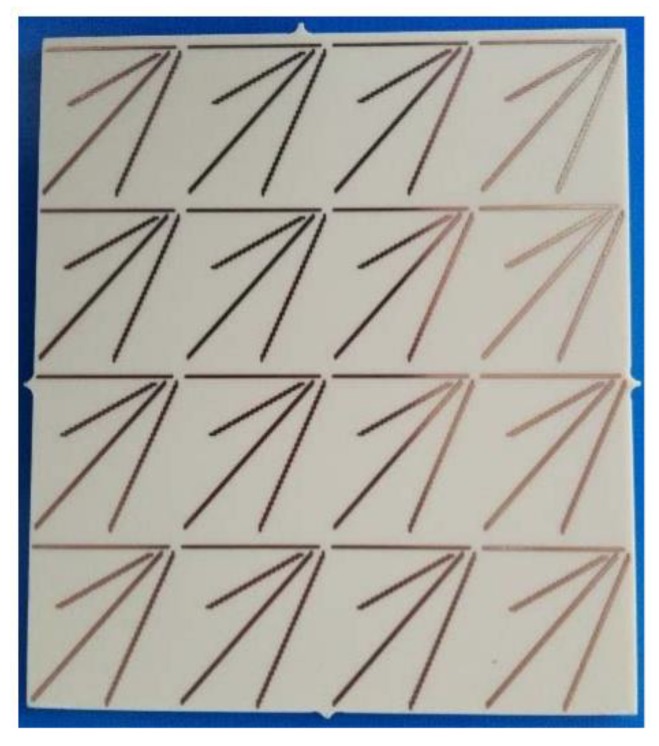
Prototype a chipless RFID sensor for angular rotation monitoring comprising a set of 4 × 4 unit cells with four-dipole elements in each one. From ref. [154].

Few papers can be found aimed at monitoring motion using chipless sensors [150,151], while angle and rotation measurement techniques are better represented [46,47,152,153,154,155,156]. An example of an angular rotation sensor is reported in Figure 20.

Finally, chipless RFID touch sensors have been proposed [157,158,159,160]. Chipless RFID touch sensors are especially attractive because they can be printed on paper [158], making them cheap and disposable, or for wearable applications [157], where they can be integrated seamlessly in textiles or garments. The working principle of the chipless touchpads is the shift or the disappearance of a resonance peak when the resonator is touched with a finger. However, compared to traditional touchpads, the main limitation of this approach is given by the surface needed by the resonators and the antenna, which increases rapidly as the number of touch locations increases. So far, six is the maximum number of single-touch events at different locations reported in the literature [160].

### 5.6. Wearables and Implants

In the case of wearable or implantable chipless RFID devices, the tag must operate in close proximity to the human body or inside the human body. In these special cases, several additional issues must be considered, including operating frequency and miniaturization constraints, as well as the dielectric influence of the human body and safety concerns. Moreover, special fabrication techniques should be employed, in order to produce a device that is conformable, flexible, biocompatible, robust and resistant to wear [60,64,65].

All the different human body tissues have a frequency-dependent dielectric constant and loss tangent which far exceed those of free space [161,162]. This has very important consequences on the chipless tag design, which must be able to operate in the presence of biological tissues, where losses usually dominate. This means, in turn, that an appropriate dielectric model of the human body must be available, and inevitably meets the problem that tissue properties may vary not only among individuals but also as a function of environmental condition or age. 

Nonetheless, this field is very promising for future applications, because the foreseen advantages of the chipless technology applied to wearables or implants are potentially enormous and of high impact for human health and wellbeing. Compared to traditional RFID, chipless tags do not need to be integrated into the wearable object but can be directly sewn or printed on clothes and garments, avoiding the connectivity o rigidity problems associated with the chips.

Several applications have already been reported for wearable chipless sensors, exploiting the low-frequency regime [63,163,164], the ultra-wide band [66,86,165,166] and the 26.5–40 GHz band [119]. Humidity sensors embedded in textiles [167] enabling sweat detection are one promising application, together with strain sensors [168] for mechanical characterization or touch sensors [157]. 

Implantable chipless tags are more challenging from the technical and normative point of view. While several implantable RFIDs have been proposed in the literature [169,170,171], reports of chipless devices are so far rather scarce. Even though the advantages of the elimination of transcutaneous wires and batteries that can be provided by this technology are evident, the complexity of the system due to wireless operation results in other drawbacks that increase the overall size of the implant [170]. The size of the antenna depends on the operation frequency, and therefore high frequencies should be preferable. Unfortunately, in biological tissues, RF losses are lower at low operating frequencies. This requires a trade-off on the designed operating frequency that will depend on the specific implant and its function. It is however clear that with the current technology available, long-range telemetry is out of the question and the reading range for in-body devices is limited to a few centimetres. This last constraint is a consequence of two technical limitations, both related to the microwave energy needed to detect the signal. Large reading ranges require more energy, especially in a lossy medium as the human body and this means that heating of the body part surrounding the implant will be unavoidable. This is not only dangerous but may also alter the sensing result. Moreover, the energy absorption is limited by the maximum authorized specific absorption (SAR) defined by the standards for given frequency ranges (e.g., in the range 100 kHz–10 GHz, the whole-body SAR should not exceed 0.4 W/kg [172]) and this allows only for limited reading ranges.

## 6. Conclusions

Chipless RFID sensors are attaining an increasingly scientific and commercial interest in several application fields related to IoT. The reason for this interest is found in their extremely low cost and their simple methods of production. While for applications in the field of tracking, chipless tags have to overcome the significant constraint of the large amount of information required, in the realization of sensors this limitation is much less severe or unimportant. 

However, many issues still have to be solved before chipless RFID sensors can reach a diffusion comparable to that of conventional RFID systems. In chemical and physical sensing, sensitivity and selectivity need to be improved with more research in the field of smart materials for sensing. For structural and environmental sensing, the limited reading distance is still an important issue. Finally, for wearable and especially for implantable sensors many constraints and limitations related to the peculiarity of the applications still hinder the practical exploitation of chipless RFID technology. On the other hand, great progress has been made in the last few years and for many low-cost applications, chipless RFIDs are already a viable alternative. Moreover, they are particularly suited for environments where traditional IC-based technologies are not exploitable, or for future green and biodegradable technologies, which are expected to grow immensely in the next years.

## Figures and Tables

**Table 1:** Dielectric properties of typical sensing materials.

Material	Dielectric Constant	Loss Factor	Sensed Parameter
Kapton HN	3.15–3.4	0.0015–0.0035	Humidity [50]
Polyvinyl alcol	≈4 [51]	Variable ^1^	Humidity [50]
Carbon nanotubes	≈−6 [52]	Very variable ^1^	Ammonia [53,54]
			CO_2_ [55]
Paper	2.4–3.4	0.08–0.3	Humidity [56]
Silicon nanowires	2–12 [57]	Variable ^1^	Humidity [58]
Water	78	0.01–0.4	Oil [58]

^1^ Depending on composition, conductivity and environment.

**Table 2:** Comparison of different techniques used for the fabrication of chipless sensors.

Fabrication Technique	Typical Resolution	Suitable for Flexible Substrates	Suitable for Mass Production	Used for Sensing Material
Microlithography	>1 μm	Partially	Yes	Yes
Inkjet printing	20–50 μm	Yes	Limited	Yes
Aerosol printing	5–10 μm	Yes	No	Yes
Screen printing	50 μm	Yes	Yes	Yes
CNC Milling	>20 μm ^1^	No	Limited	No
Embroidery	>0.5 mm	Yes	Limited	No
Drop casting	-	Partially	Yes	Yes
Thermocompressive bonding	>20 μm ^1^	Partially	Limited	Yes

^1^ Strongly dependent on dimensions and process parameters.
